# Next-Generation Sequencing in Melioidosis: Enhancing Diagnosis, Epidemiology and Antimicrobial Resistance Surveillance

**DOI:** 10.3390/diagnostics16162613

**Published:** 2026-08-18

**Authors:** Hua Wu, Pei Zhang, Shijia Li, Huimin Zhao

**Affiliations:** 1 Department of Clinical Laboratory, Hainan Women and Children’s Medical Center, Haikou 570206, China; 2 Department of Clinical Laboratory, Hainan General Hospital, Haikou 570311, China; 3 Affiliated Hainan Hospital, Hainan Medical University, Haikou 570102, China

**Keywords:** melioidosis, *Burkholderia pseudomallei*, next-generation sequencing, diagnosis, genomic epidemiology, antimicrobial resistance

## Abstract

Melioidosis, caused by *Burkholderia pseudomallei*, is a severe infectious disease with high mortality. Diagnostic delays due to conventional culture and serology limitations impact patient outcomes. This narrative review synthesizes evidence on next-generation sequencing (NGS) in melioidosis. NGS technologies encompass two main applications: metagenomic NGS (mNGS), which enables culture-independent detection directly from clinical samples, and whole-genome sequencing (WGS), which provides outbreak tracing and source attribution from cultured isolates. Resistance profiling detects antimicrobial resistance (AMR) determinants (e.g., *penA* mutations) to guide therapy. Recent 2025–2026 studies highlight new applications, including direct pathogen genome recovery from environmental samples. Despite cost and standardization challenges, integrating NGS into clinical workflows holds promise for improving melioidosis management, especially in resource-limited settings.

## 1. Introduction

Melioidosis is a formidable global health threat, with an estimated 165,000 cases and 89,000 deaths annually, primarily in Southeast Asia and northern Australia [1]. The causative agent, *Burkholderia pseudomallei*, is a soil-dwelling bacterium that infects humans through percutaneous inoculation, inhalation, or ingestion, leading to clinical manifestations ranging from localized abscesses to fulminant septicemia. The disease is often termed the “great mimicker” due to its nonspecific presentation, which overlaps with various other infectious and non-infectious conditions, complicating timely diagnosis and management [2]. Established risk factors for melioidosis include diabetes mellitus (increasing risk 5- to 10-fold), male sex (2:1 male-to-female ratio), occupational exposure (particularly rice farming), and rural residence in endemic areas [1].

The diagnostic cornerstone remains bacterial culture, which typically requires 2–3 days for visible growth, with full identification and susceptibility testing extending to 3–7 days depending on laboratory capacity. The estimated culture sensitivity is 60–70% [3]. The sensitivity of culture is limited by slow bacterial growth and frequent misidentification with closely related species such as *Burkholderia cepacia*, *Burkholderia thailandensis*, and *Pseudomonas* spp., especially in laboratories unfamiliar with *B. pseudomallei*. Serological tests such as the indirect hemagglutination assay suffer from variable sensitivity and specificity, cross-reactivity, and lack of standardization [4,5]. A recent meta-analysis confirmed suboptimal accuracy of non-culture methods, underscoring the urgent need for improved diagnostics [6].

Despite increasing recognition of melioidosis as a global health threat, current estimates of its disease burden likely underestimate the true scope. The disease remains substantially underreported in many endemic regions due to limited diagnostic capacity, lack of surveillance infrastructure, and the nonspecific clinical presentation that mimics other febrile illnesses. Moreover, the geographic distribution of *B. pseudomallei* is expanding, with climate change and extreme weather events increasingly recognized as drivers of environmental dispersal and disease emergence. These developments, together with the accelerating pace of NGS technology innovation, make an updated synthesis timely. While previous reviews have addressed NGS applications in infectious diseases broadly, a focused critical appraisal of NGS in melioidosis management has not been undertaken since the recent advances in real-time sequencing and hybrid assembly approaches. This review aims to fill this gap by critically evaluating the current evidence, identifying unresolved challenges, and proposing practical pathways for implementation in endemic and resource-limited settings.

The clinical management of melioidosis is further complicated by the pathogen’s intrinsic resistance to multiple antibiotics and its ability to form biofilms, contributing to persistent infections and relapse even after prolonged antimicrobial therapy. Despite prolonged therapy, relapse rates of 5–10% are reported, primarily associated with incomplete treatment adherence, deep-seated infection foci, and emerging antimicrobial resistance (AMR) [3].

Next-generation sequencing (NGS) represents a transformative technology for clinical microbiology. This review synthesizes evidence on how next-generation sequencing (NGS) technologies are addressing the critical challenges of rapid diagnosis, transmission dynamics, and antimicrobial resistance (AMR) in melioidosis. We distinguish two complementary applications: (1) metagenomic NGS (mNGS), in which total nucleic acids from clinical specimens are sequenced directly without prior culture, enabling rapid, unbiased pathogen detection; and (2) whole-genome sequencing (WGS) of cultured isolates (typically performed on NGS platforms), which provides the high-resolution genomic data needed for phylogenetic analysis, outbreak tracing, and comprehensive resistance gene identification.

## 2. Search Strategy

This narrative review was conducted by searching PubMed, Web of Science, and Scopus databases for articles published between January 2015 and 2026, using combinations of search terms including: “*Burkholderia pseudomallei*”, “melioidosis”, “next-generation sequencing”, “whole-genome sequencing”, “metagenomics”, “diagnosis”, “antimicrobial resistance”, and “genomic epidemiology.” Additional relevant references were identified through manual screening of reference lists. Titles and abstracts were screened for relevance to the three thematic areas: diagnosis, genomic epidemiology, and antimicrobial resistance. Full-text articles were assessed against the following inclusion criteria: (1) original research or systematic reviews on NGS applications in *B. pseudomallei* or melioidosis; (2) studies reporting primary data on diagnostic accuracy, genomic epidemiology, outbreak tracing, environmental surveillance, or antimicrobial resistance profiling; (3) articles published in English between January 2015 and 2026. Studies not directly addressing NGS applications, opinion pieces without primary data, and non-English articles were excluded. The studies highlighted in this review were selected based on their novelty, methodological rigor, and direct relevance to the three thematic areas.

## 3. NGS for Accelerating Diagnosis

The prolonged turnaround time of culture, a critical driver of treatment delays, necessitates faster diagnostic approaches. mNGS sequences all nucleic acids in a clinical sample without prior culturing, enabling unbiased pathogen detection. Two main NGS strategies are available for diagnosis: targeted NGS (tNGS) and mNGS. tNGS uses amplicon or probe capture to enrich for specific genomic regions of interest, such as *B. pseudomallei*-specific markers or resistance genes. This approach offers higher sensitivity for targeted organisms, lower cost, and faster turnaround, making it suitable for surveillance and known resistance profiling. In contrast, mNGS involves untargeted sequencing of all nucleic acids, enabling detection of any pathogen, including unexpected or novel species, but is challenged by high background of host DNA, particularly in blood and tissue samples [7]. The key differences between these approaches are summarized in Table 1. Additionally, clinical specimens often contain complex biological matrices (e.g., mucin, cellular debris, or PCR inhibitors) that can interfere with library preparation and reduce sequencing efficiency, necessitating specialized protocols for nucleic acid extraction and host depletion.

**Figure 1:**
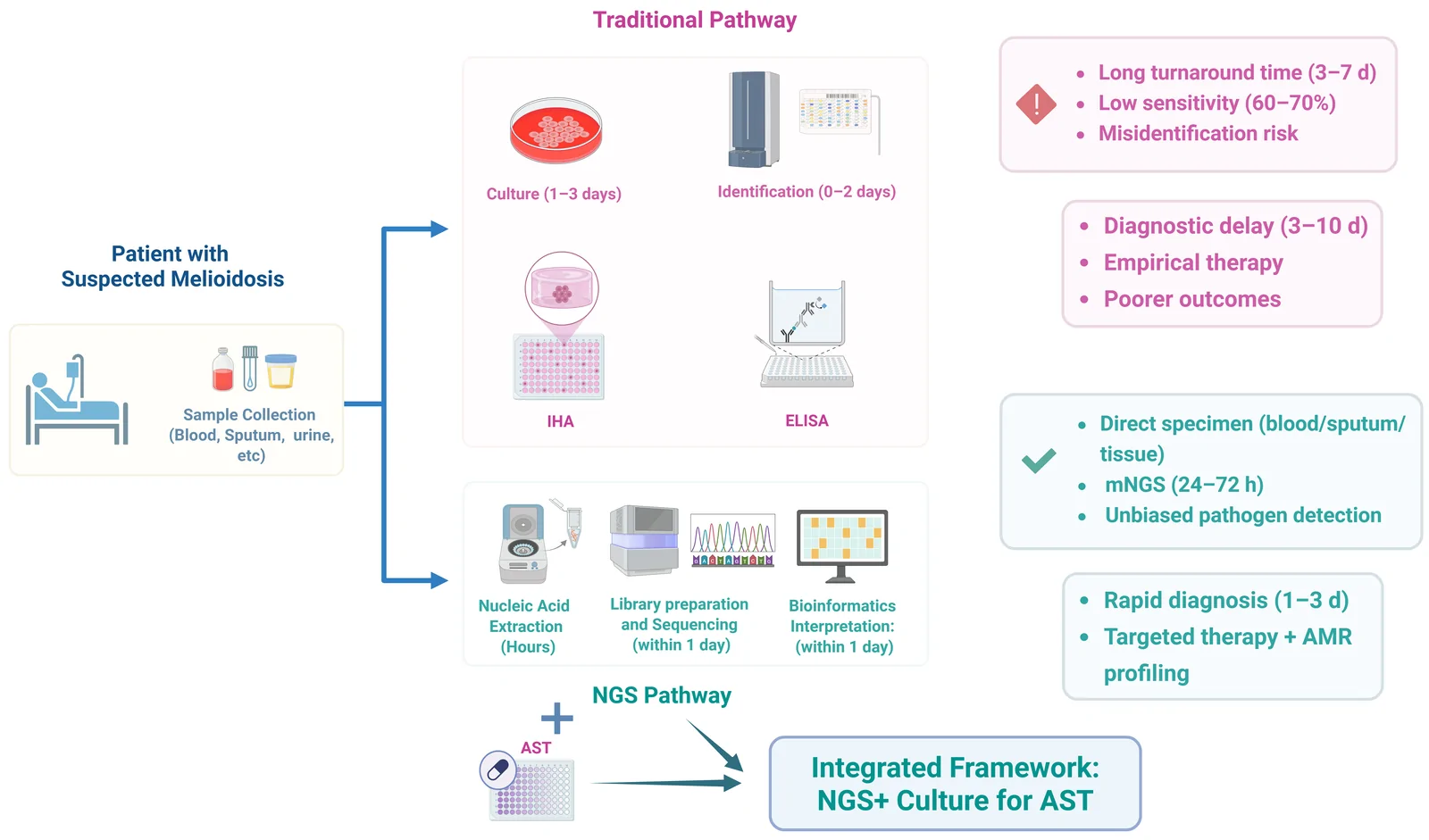
Comparative diagnostic pathways for melioidosis: Traditional vs. NGS-based approaches. This schematic illustrates the critical divergence between conventional diagnostic methods and next-generation sequencing (NGS). The traditional pathway is hindered by prolonged culture times (visible growth typically 2–3 days, with full identification and AST extending to 3–7 days) and serological limitations, leading to diagnostic delays and empirical therapy. The NGS-based pathway enables direct, rapid pathogen detection from clinical samples, significantly shortening the time to diagnosis and providing simultaneous access to genomic data for resistance profiling. NGS complements traditional methods, forming an integrated diagnostic framework that combines speed with reliability.

The comparative advantages of NGS over conventional diagnostic workflows are illustrated in Figure 1. In melioidosis, mNGS has successfully identified *B. pseudomallei* from clinical samples including blood, sputum, and tissue, significantly reducing diagnostic turnaround time from days to hours. In a sepsis study from Cambodia, the combination of NGS and traditional diagnostics identified a pathogen in approximately 50% of cases [8]; this percentage can vary substantially depending on patient population, sample type, timing of collection, and prior antibiotic exposure. For pediatric populations in Mali where melioidosis was previously unrecognized, WGS of blood culture isolates revealed substantial disease burden and high mortality, underscoring the need for improved rapid diagnostics [9]. A case report from China further illustrated the utility of mNGS in diagnosing chronic splenic melioidosis in a patient with fever of unknown origin, where conventional cultures remained negative [10].

Recent 2026 evidence further supports the diagnostic value of NGS. A case in Beijing demonstrated that mNGS of bronchoalveolar lavage fluid rapidly identified *B. pseudomallei* in a 6-year-old imported melioidosis case from Thailand, enabling timely targeted therapy and confirming the pathogen as sequence type 395 (ST395), a type not previously reported in China [11]. Similarly, a 2026 case from Haikou, China, reported that mNGS identified *B. pseudomallei* ST271 from bronchoalveolar lavage fluid in a patient with rapidly progressive cavitary pneumonia and reversible hepatic injury, facilitating early targeted therapy and favorable recovery [12].

While such case reports provide compelling illustrations of the diagnostic potential of mNGS, it is important to distinguish between evidence of feasibility and evidence of clinical utility. Case reports demonstrate that mNGS can detect *B. pseudomallei* in situations where conventional methods fail; however, they do not establish that mNGS improves patient outcomes, reduces antibiotic use, or is cost-effective at a population level. The limited generalizability of individual case reports, which are subject to publication bias and often describe unusual or complex scenarios, must be acknowledged. The clinical adoption of mNGS for melioidosis diagnosis therefore requires stronger evidence from prospective cohort studies and controlled evaluations, which currently remain sparse.

**Figure 2:**
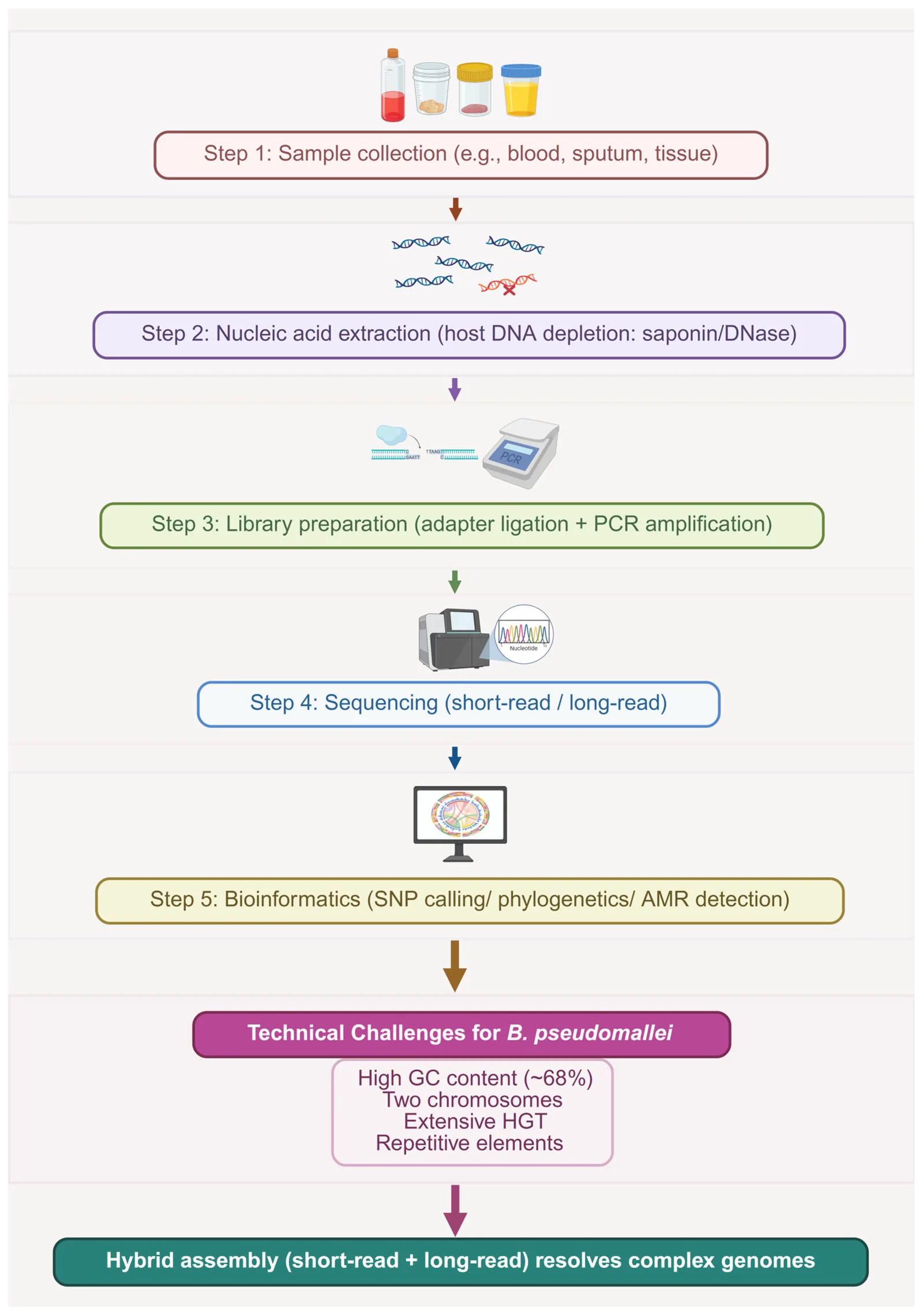
NGS workflow for *B. pseudomallei* and associated technical challenges. The workflow proceeds vertically from sample collection (e.g., blood, sputum, tissue) through nucleic acid extraction with host DNA depletion using saponin or DNase, library preparation via adapter ligation and PCR amplification, sequencing using short-read and long-read platforms, and bioinformatics analysis including SNP calling, phylogenetics, and AMR detection. *B. pseudomallei* presents specific technical challenges that complicate assembly: high GC content (~68%), a two-chromosome genome (~7.2 Mb), extensive horizontal gene transfer, and repetitive elements. These challenges require hybrid approaches combining short-read and long-read sequencing for complete genome reconstruction. The primary outputs for epidemiological and clinical applications are SNP-based phylogenetic analysis and AMR gene detection.

The sequencing workflow and the specific technical challenges posed by the *B. pseudomallei* genome are illustrated in Figure 2.

From a technical perspective, the success of mNGS depends heavily on efficient removal of host-derived nucleic acids. In samples like blood or tissue, the overwhelming abundance of human DNA can drastically reduce the sequencing depth available for microbial reads. Protocols incorporating saponin-based host depletion or DNase treatment have been shown to improve sensitivity for low-abundance pathogens [13]. Additionally, the choice of sequencing platform influences turnaround time and accuracy. Short-read Illumina platforms provide high accuracy (>99.9%) ideal for single nucleotide polymorphism detection, while long-read Oxford Nanopore Technology (ONT) offers real-time sequencing and portability, albeit with higher raw error rates that require computational correction.

The complex genome of *B. pseudomallei*, with its two chromosomes, high GC content (~68%), extensive repetitive elements, and frequent horizontal gene transfer, poses specific challenges for sequencing and assembly, particularly for short-read platforms. Weigl and colleagues recently developed an optimized ONT-based core genome MLST workflow for *B. pseudomallei* that addresses some of these challenges, integrating high resolution with enhanced cost-efficiency for rapid diagnostics [14]. For complex genomes such as *B. pseudomallei*, hybrid approaches combining short-read (Illumina) and long-read (Oxford Nanopore or PacBio) sequencing are often required to obtain complete, accurate genome assemblies.

Beyond pathogen detection, NGS-based diagnostic approaches also enable simultaneous identification of AMR genes directly from clinical samples, providing early warning of potential treatment challenges. For example, metagenomic sequencing can detect resistance determinants such as *penA* mutations or *amrR* deletions even before culture results are available, allowing clinicians to anticipate possible resistance and adjust empirical therapy accordingly. Unlike multidrug-resistant Enterobacteriaceae, where resistance genes are often carried on plasmids, acquired resistance in *B. pseudomallei* is predominantly chromosomal, arising through point mutations, gene duplications, or promoter alterations. This chromosomal nature has important implications for sequencing: detection of low-frequency resistance mutations requires sufficient sequencing depth, and it limits the utility of plasmid-centric resistance databases. Clinically, chromosomal resistance mutations are stable within clonal lineages, underscoring the importance of genomic surveillance to monitor emerging resistance patterns.

The availability of NGS-based assays in endemic settings varies widely. In well-resourced reference laboratories such as those at MORU in Thailand or the Menzies School in Australia, WGS is increasingly routine for epidemiological surveillance and outbreak investigation. However, in most district-and provincial-level hospitals across Southeast Asia and other endemic regions, NGS remains largely inaccessible due to cost, infrastructure requirements, and limited bioinformatics capacity. Targeted PCR-based assays (e.g., TTS1-orf2 qPCR) and LAMP are currently the most feasible molecular diagnostics at the frontline level. Regional sequencing hubs, such as those supported by the Wellcome Trust in Thailand, offer a middle-ground solution, allowing samples from peripheral sites to be referred for genomic analysis when clinically or epidemiologically indicated.

It is important to recognize that rapid molecular methods including qPCR (targeting TTS1-*orf2* with 79.2% sensitivity and 99% specificity) and LAMP assays remain faster (2–4 h) and more practical for frontline use in endemic settings [15]. These assays are well-suited for initial screening in district hospitals and reference laboratories where NGS infrastructure is unavailable. NGS is best positioned as a second-tier tool for cases where initial testing is negative despite high clinical suspicion, for comprehensive resistance profiling, and for epidemiological investigation [16]. The circumstances under which NGS offers a clear advantage over PCR-based methods include (1) suspected polymicrobial infections requiring broad pathogen detection, (2) cases with negative PCR results but strong clinical suspicion of melioidosis, and (3) situations requiring simultaneous resistance profiling. For routine diagnostic use in well-resourced settings, NGS may also be considered, but its cost-effectiveness and clinical impact require further validation.

The key distinctions between these three application domains are summarized in Table 2, which provides a comparative overview of their objectives, approaches, and challenges.

## 4. Genomic Epidemiology: Tracing Transmission and Environmental Sources

**Figure 3:**
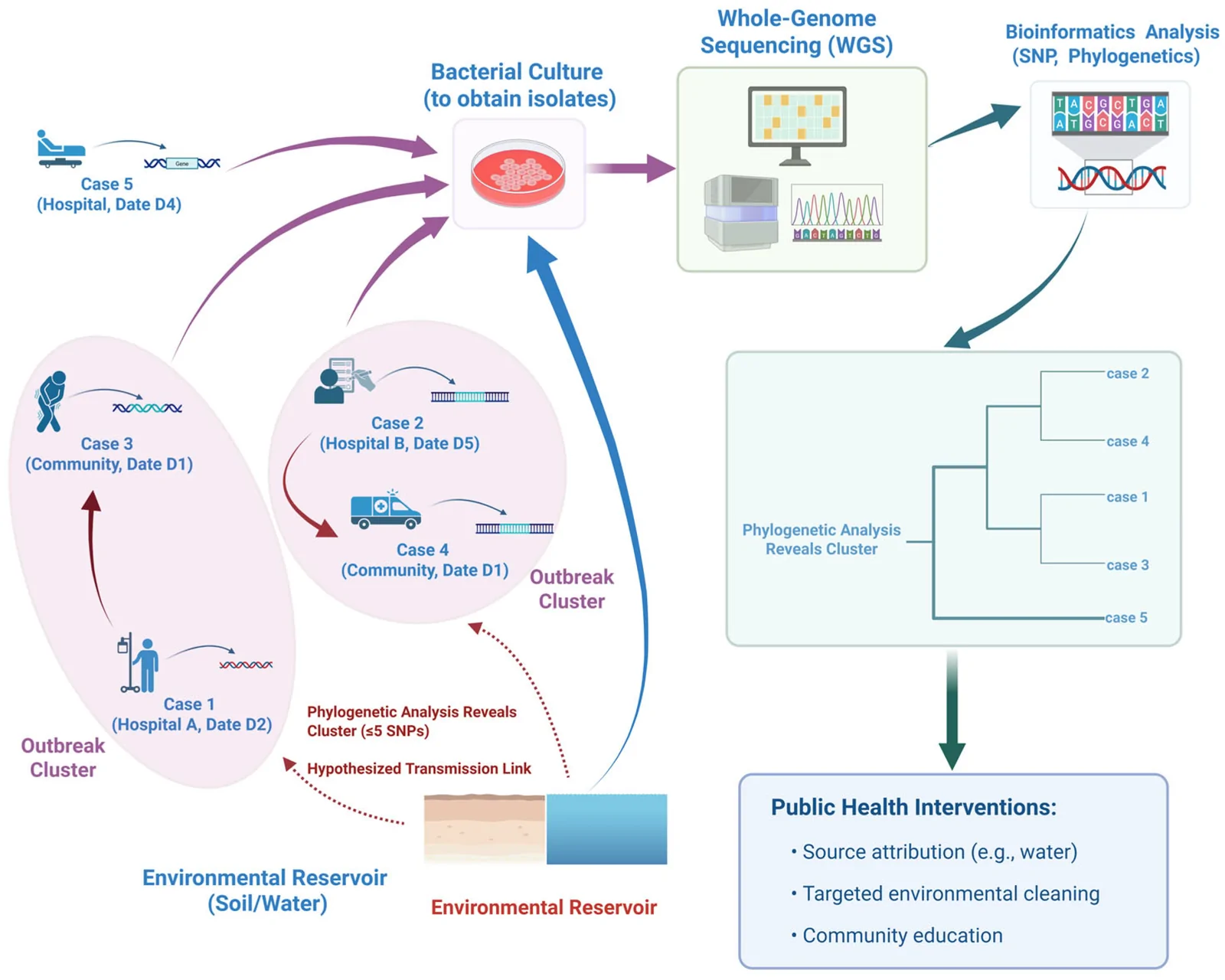
Application of NGS in genomic epidemiology and outbreak tracing of melioidosis. This schematic illustrates the workflow for linking clinical cases to environmental sources using whole-genome sequencing (WGS). Clinical isolates and environmental samples are sequenced and compared through phylogenetic analysis to identify outbreak clusters and transmission links. Dashed arrows indicate hypothesized transmission from environmental sources, while solid lines connect cases with high genomic similarity. The threshold of ≤5 SNPs, while commonly used as a rule of thumb for recent transmission in many bacterial pathogens, should be interpreted with caution for *B. pseudomallei*, as the genomic evolutionary rate and recombination frequency may vary by lineage and geographical setting.

Building on the diagnostic applications discussed in Section 3 and summarized in Table 2, this section focuses on the role of WGS in genomic epidemiology. The workflow for linking clinical isolates to outbreak detection and source attribution is illustrated in Figure 3.

Despite its power, WGS-based epidemiological inference is subject to important methodological limitations. The interpretation of SNP thresholds for transmission linkage, commonly set at ≤5 SNPs, must be approached with caution for *B. pseudomallei*, as the genomic evolutionary rate and recombination frequency may vary substantially across lineages and geographical regions. Recombination events can generate SNP patterns that mimic clonal transmission, potentially leading to false-positive linkage between unrelated cases. Conversely, regions with high genomic diversity may require lineage-specific SNP thresholds to avoid underestimating transmission. The genomic diversity of *B. pseudomallei* across Southeast Asia, Australia, and other endemic regions further complicates phylogenetic inference, as reference genomes from one region may not be optimal for mapping reads from another. These challenges underscore the need for integrating WGS data with detailed epidemiological metadata, including patient travel histories, spatial clustering, and temporal relationships, to confidently distinguish between true transmission, relapse, and reinfection. Comparative studies using standardized analytical pipelines and validated reference databases are needed to improve the reproducibility and comparability of WGS-based epidemiological investigations.

WGS of cultured *B. pseudomallei* isolates has become a powerful tool for high-resolution molecular epidemiology, providing resolution far exceeding that of traditional typing methods such as multilocus sequence typing (MLST) or pulsed-field gel electrophoresis (PFGE) [17,18]. However, WGS does not replace standard epidemiological investigation; rather, it complements and refines it by providing molecular evidence to support or refute transmission hypotheses. Combining WGS data with detailed patient travel histories, environmental sampling, and geospatial analysis yields the most robust conclusions about transmission chains and source attribution [17]. By comparing single nucleotide polymorphisms across entire genomes, WGS can accurately distinguish between relapse and re-infection and elucidate fine-scale transmission chains.

A pivotal application is linking human infections to environmental reservoirs. Webb and colleagues demonstrated this by genomically linking *B. pseudomallei* from individual human cases to isolates from targeted environmental sampling in northern Australia, providing concrete evidence for specific environmental sources of infection [17]. Similarly, genomic analysis of *B. pseudomallei* isolates from Malaysia revealed distinct phylogeographic clusters corresponding to different regions, providing insights into local transmission dynamics [24]. In Colombia, WGS of isolates from soil and water demonstrated that closely related strains are often confined to specific geographical areas [25].

The key epidemiological studies reviewed reveal notable differences in study design, geographic scope, and sequencing approach. The Darwin Prospective Melioidosis Study [26], a 30-year prospective investigation, has provided longitudinal genomic data linked to clinical and environmental metadata in northern Australia. In contrast, large-scale cross-sectional studies from Thailand [18] and Malaysia [24] have used population-based sampling to identify phylogeographic clustering and environmental drivers of transmission. While these studies consistently demonstrate the power of WGS for outbreak tracing, their differing designs, including prospective versus retrospective, population-based versus outbreak-focused, and varying sequencing depth and analytical pipelines, limit direct comparability. Harmonization of sequencing protocols and analytical pipelines would facilitate meta-analyses and cross-regional comparisons, a priority for future surveillance efforts.

Beyond outbreak investigations, WGS combined with geospatial analysis enhances understanding of pathogen spread. Genomic surveillance of *B. pseudomallei* in soil and water reservoirs has revealed substantial genetic diversity and spatial structuring, informing environmental sampling strategies and risk mapping [27]. A large-scale genomic analysis of 1391 *B. pseudomallei* isolates from northeast Thailand identified three dominant lineages, each with unique gene sets potentially enhancing bacterial fitness in the environment, and highlighted the influence of environmental factors such as terrain slope, altitude, and river direction on geographical dispersal [18].

Recent 2025–2026 research has further advanced the field. A study using shotgun metagenomics successfully reconstructed a near-complete metagenome-assembled genome directly from an aromatherapy spray that caused a U.S. outbreak in 2021, demonstrating that metagenomics can recover high-quality pathogen genomes from complex samples despite the presence of related microbes, supporting outbreak investigations and enhancing public health surveillance [28].

In parallel, genomic surveillance in non-endemic regions has gained increasing importance. Whole-genome sequencing and comparative genomics of two travel-related melioidosis cases confirmed in Hungary between 2008 and 2024 identified a novel sequence type ST1643 and ST1051, both clustering within the Asian clade. A systematic review accompanying this study identified 195 European melioidosis cases between 1980 and 2025, most originating from Southeast Asia, with pneumonia, septic form, and abscess as the predominant presentations [29]. These findings highlight the need for improved clinical awareness, genomic surveillance, and diagnostic preparedness in non-endemic regions, as global travel and climate change expand the distribution of melioidosis.

Another genomic analysis confirmed the unprecedented presence of an Asian clone (ST-562) in Darwin, northern Australia, with the incidence of ST-562 infection steadily rising since 2005, now representing a common strain in the region, indicating recent introduction of this clone [30].

Environmental surveillance using NGS is also evolving beyond WGS of cultured isolates. Amplicon sequencing targeting 16S rRNA or species-specific genes can monitor microbial community composition and detect *B. pseudomallei* in water and soil samples without prior culture. Quantitative metagenomic approaches allow absolute quantification of antibiotic resistance genes and pathogenic bacteria, providing a more accurate assessment of environmental contamination levels and potential health risks [31]. For *B. pseudomallei* specifically, longitudinal sampling coupled with NGS-based community profiling can detect emerging strains or resistance profiles in environmental reservoirs, informing early warning systems for outbreak risks. For instance, studies in northern Australia have demonstrated the value of repeated environmental sampling to identify persistent *B. pseudomallei* hotspots in soil and water sources, with genomic data linking these reservoirs to clinical cases [17]. Similar approaches are being developed in Thailand and other endemic regions as part of ongoing surveillance efforts. Antimicrobial resistance in *B. pseudomallei* is less widespread than in many Enterobacteriaceae, but it remains a clinically significant concern. Acquired resistance to first-line agents (ceftazidime, meropenem, and trimethoprim-sulfamethoxazole) is rare in treatment-naïve isolates, with global surveillance studies reporting rates of <1–2% for ceftazidime resistance in most endemic regions. However, resistance can emerge during treatment, particularly in patients with deep-seated infections, prolonged therapy, or suboptimal adherence, with relapse isolates showing acquired resistance in 5–10% of cases. The increasing detection of novel resistance mutations and the occasional report of acquired carbapenemase genes (e.g., blaNDM, blaOXA-48) underscore the importance of ongoing genomic surveillance.

Together, these challenges highlight that while WGS has transformed epidemiological investigations of melioidosis, its conclusions must be interpreted in the context of methodological limitations. Future studies should prioritize the development of validated, lineage-specific SNP thresholds, the use of hybrid assembly approaches to resolve recombination-prone regions, and the integration of genomic data with detailed epidemiological metadata to improve the accuracy and robustness of transmission analyses.

## 5. Profiling Antimicrobial Resistance Through Genomic Insights

As outlined in Table 2, antimicrobial resistance profiling represents the third major application domain of NGS in melioidosis. NGS revolutionizes AMR management in melioidosis by enabling rapid, comprehensive genotypic resistance profiling. Unlike conventional phenotypic testing, which requires 7–10 days, NGS can detect resistance determinants directly from sequence data, dramatically shortening the time to actionable results. This is critical for guiding therapy against key agents like ceftazidime.

Madden and colleagues highlighted the utility of NGS for comprehensive AMR detection from *B. pseudomallei* genomes, developing the ARDaP tool for accurate prediction of AMR determinants from WGS data [20]. The tool has demonstrated high concordance with phenotypic susceptibility testing for multiple antibiotics, including ceftazidime, meropenem, and trimethoprim-sulfamethoxazole. Similarly, publicly available databases such as ResFinder and AMRFinderPlus can be used for *B. pseudomallei* resistance gene identification [20], although they require careful curation due to species-specific resistance mechanisms.

Specific mutations have been identified as key resistance mechanisms. The P174L point mutation in the *penA* gene confers ceftazidime resistance in Chinese isolates [21]. Meropenem resistance has been associated with amrR deletions leading to AmrAB-OprA efflux pump overexpression [31]. NGS can also detect horizontally acquired carbapenemase genes, such as the rare coexistence of blaNDM and *blaOXA-48* in a clinical *B. pseudomallei* isolate [22].

Recent genomic studies from Thailand have substantially expanded our understanding of ceftazidime resistance mechanisms. A 2025 study identified a novel A172T mutation in PenA, along with palindromic GC-rich repeat sequences that facilitate *penA* duplication and amplification, representing a newly discovered mechanism driving ceftazidime resistance. Frequent gene duplication and amplification of *penA*, likely driven by these palindromic GC-rich repeat sequences, highlights the urgent need for rapid diagnostics and optimized treatment strategies [32].

Further illuminating within-host resistance evolution, a 2025 paired isolate study from Thailand examined *B. pseudomallei* isolates collected before and after 23 days of intravenous ceftazidime therapy in a pediatric patient with acute pyelonephritis. WGS revealed no known ceftazidime-resistance mutations in *penA* coding sequences but identified a G(-78)A mutation upstream of *penA*, associated with increased promoter activity and β-lactam resistance. Notably, minimal inhibitory concentrations for ceftazidime increased from 1 μg/mL to 32 μg/mL, while values remained within the susceptible range by CLSI breakpoint criteria during early detection, underscoring the importance of rapid molecular diagnostics for timely antibiotic adjustments [33].

Longitudinal genomic studies of *B. pseudomallei* during treatment have also documented other forms of within-host evolution of resistance. A case report from Australia documented acquisition of a *penA* mutation conferring ceftazidime resistance during prolonged therapy [34]. A complete genome sequencing study of a ceftazidime-resistant *B. pseudomallei* strain (490f) from a treatment failure case in northeast Thailand revealed a 150 kb region on the small chromosome with high coverage and complex repeats, which were manually resolved using Oxford Nanopore and Illumina sequencing techniques [35].

The predictive accuracy of NGS-based resistance genotyping for *B. pseudomallei* is improving. For ceftazidime, the presence of known *penA* mutations (e.g., P167S, P174L, A172T) predicts resistance with high sensitivity (>95%) and specificity (>95%) based on analyses of clinical isolates with paired phenotypic and genotypic data [20,21]. For meropenem, *amrR* loss-of-function mutations are strongly correlated with resistance, though the precise sensitivity and specificity require further validation due to the limited number of meropenem-resistant clinical isolates available for study [23]. However, challenges remain for drugs like trimethoprim-sulfamethoxazole, where resistance mechanisms are more heterogeneous and include mutations in folP, folA, and dfrA genes, as well as efflux pump overexpression. Continued refinement of resistance gene databases and validation against phenotypic data are essential to realize the full potential of NGS-guided therapy.

However, several limitations of genotype-based resistance prediction must be acknowledged. Discordance between genotype and phenotype can occur: not all mutations identified by sequencing confer clinically significant resistance, and resistance may be mediated by mechanisms not captured by current databases, such as gene amplification, promoter mutations, or efflux pump overexpression. For trimethoprim-sulfamethoxazole, resistance mechanisms are particularly heterogeneous, involving multiple genes (*folP*, *folA*, *dfrA*) and regulatory pathways, making phenotype prediction from genotype more challenging [23]. The limited availability of clinical isolates with paired phenotypic and genotypic data (particularly for meropenem and trimethoprim-sulfamethoxazole) restricts the validation of prediction models. Additionally, the chromosomal nature of resistance in *B. pseudomallei* means that resistance is stable within lineages but also limits the ability to detect horizontal acquisition of resistance genes, a key advantage for plasmid-mediated resistance in Enterobacteriaceae [20]. Prospective clinical validation of genotype-based predictions across diverse geographic regions, coupled with standardized phenotypic testing, is urgently needed to define the sensitivity and specificity of NGS-based resistance predictions in real-world clinical settings.

**Figure 4:**
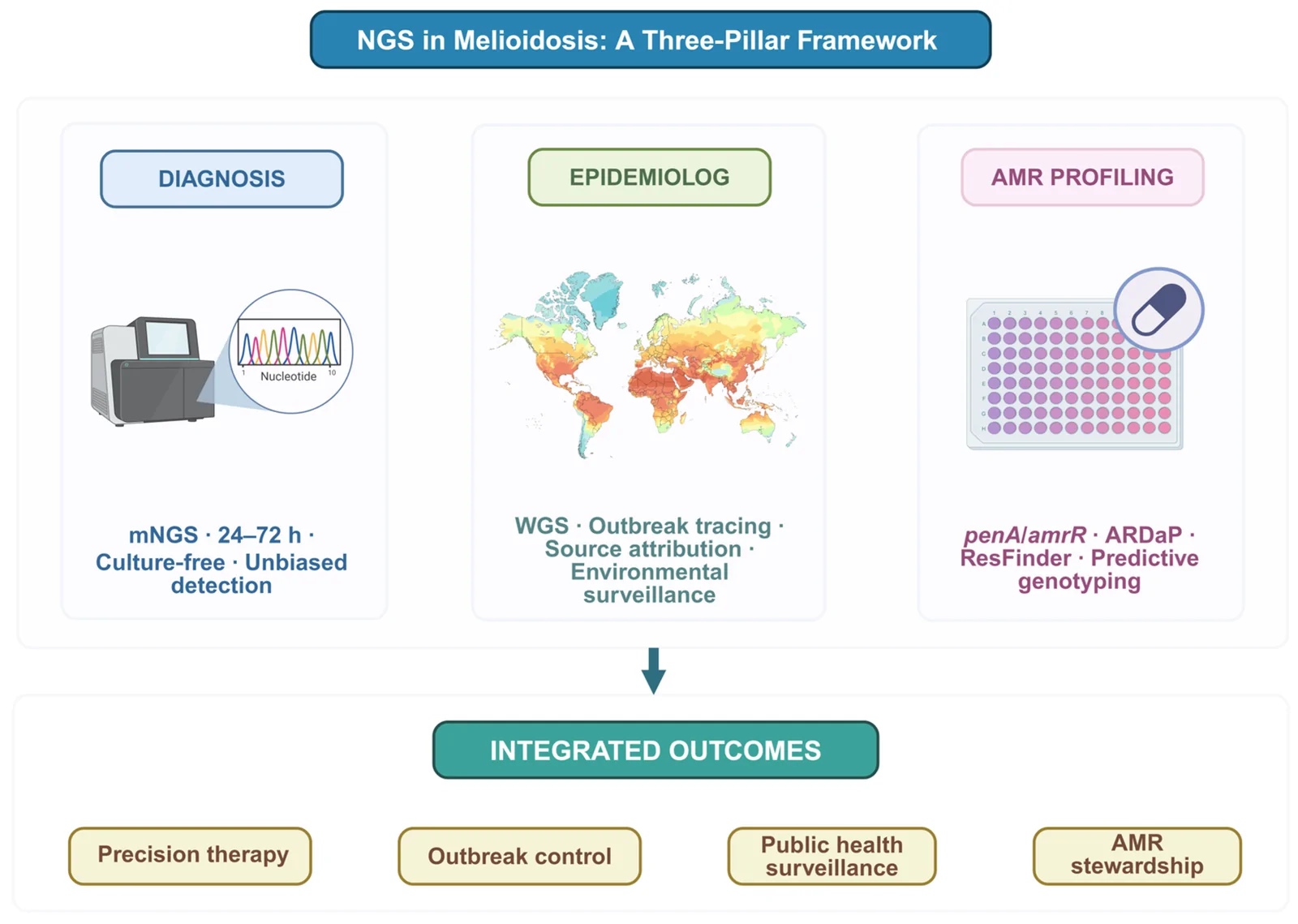
Integrated framework of NGS applications in melioidosis management. NGS technologies address three complementary pillars. The first pillar is diagnosis, where metagenomic NGS (mNGS) enables rapid, culture-independent detection directly from clinical specimens. The second pillar is epidemiology, where whole-genome sequencing (WGS) of cultured isolates provides high-resolution data for outbreak tracing, source attribution, and environmental surveillance. The third pillar is antimicrobial resistance profiling, where resistance genotyping detects key determinants such as *penA* mutations and *amrR* deletions to guide targeted therapy. These three pillars converge to support precision therapy, outbreak control, public health surveillance, and antimicrobial stewardship in melioidosis-endemic settings.

The three complementary applications of NGS in melioidosis, namely diagnosis, epidemiology, and antimicrobial resistance profiling, are integrated in a conceptual framework illustrated in Figure 4, which highlights how these pillars converge to support precision therapy, outbreak control, and public health surveillance.

## 6. Challenges and Pragmatic Pathways for Implementation

The integration of NGS into routine melioidosis management faces significant hurdles, especially in resource-limited endemic regions.

**Cost and infrastructure.** The initial investment in sequencing platforms, reagents, and computational infrastructure remains substantial, with per-sample costs substantially higher than conventional methods [16]. However, the decreasing cost of sequencing, particularly with nanopore platforms, and the development of simplified library preparation kits are gradually improving affordability [16]. While the direct per-sample cost of NGS remains substantially higher than conventional methods, it is important to realistically assess potential downstream cost savings. In confirmed melioidosis cases, the ability to tailor therapy based on resistance genotype may reduce the use of broad-spectrum antibiotics, but the overall impact on individual patient costs remains uncertain and likely varies by setting. The most clearly demonstrated cost-effectiveness of NGS in melioidosis to date lies in outbreak investigation and public health surveillance, where early detection of transmission clusters can trigger targeted interventions that prevent multiple cases. Formal cost-effectiveness analyses in endemic settings, considering both clinical and public health outcomes, are urgently needed to guide resource allocation.

**Standardization.** Lack of standardized protocols for sample processing, sequencing, and bioinformatics analysis can lead to inter-laboratory variability. For mNGS, standardized methods for host DNA depletion and contamination-aware bioinformatics are particularly vital [7]. Efforts such as the NGS Quality Initiative are working to establish consensus guidelines for clinical laboratories [36].

**Data interpretation and expertise.** Interpreting NGS data, especially distinguishing true pathogens from background noise in mNGS, requires specialized bioinformatics expertise and careful clinical correlation [37]. The development of user-friendly, automated analysis pipelines (e.g., cloud-based platforms with curated reference databases) is critical to lower the barrier for routine clinical use.

**Ethical and data governance considerations.** Genomic data contain sensitive patient information, and robust privacy-preserving technologies (e.g., secure multi-party computation, federated learning) are needed to enable data sharing while minimizing privacy risks [38]. Legal frameworks such as GDPR in Europe and POPIA in South Africa impose strict controls on genomic data access and sharing, and similar regulations are needed in endemic regions.

International frameworks for genomic data sharing, such as the Global Alliance for Genomics and Health (GA4GH), have established standards and policies that can guide the development of pathogen genomic surveillance networks. While GA4GH has a broader focus encompassing human and cancer genomics, its principles of data interoperability, privacy protection, and ethical governance are directly applicable to microbial genomics. For melioidosis specifically, the International Melioidosis Network (IMN) and the biennial World Melioidosis Congress provide existing platforms for collaboration and data sharing, which could serve as foundations for more formalized genomic surveillance initiatives.

**Biosafety and biosecurity.** As a Tier 1 select agent, *B. pseudomallei* requires enhanced biosafety measures. International guidelines recommend handling of confirmed *B. pseudomallei* isolates within a BSL-3 facility, yet studies suggest the risk of laboratory-acquired melioidosis may be low even when BSL-2 precautions are followed [39]. In endemic resource-limited settings, many diagnostic laboratories routinely perform initial culture and DNA extraction in BSL-2 facilities using standard precautions. To balance safety with practicality, NGS workflows should be designed to minimize handling of viable pathogen material, with DNA extraction as the primary point of BSL-2 handling and sequencing conducted in BSL-1 conditions.

To address these challenges, pragmatic integration strategies include: (1) developing targeted NGS panels focusing on *B. pseudomallei* and key resistance genes as a cost-effective middle ground; (2) establishing regional sequencing hubs to concentrate resources and expertise; (3) investing in capacity building and user-friendly, automated bioinformatics pipelines; (4) maintaining complementary use of rapid methods (qPCR, LAMP, CRISPR-based assays) for frontline screening; and (5) fostering international data-sharing collaborations with clear governance frameworks. A recent CRISPR-based detection method (CRISPR-BP34) has been benchmarked for point-of-care melioidosis detection in low- and middle-income countries, demonstrating the potential of these technologies beyond traditional laboratory settings [40].

## 7. Future Perspectives

Looking ahead, several priority areas will determine the extent to which NGS transforms melioidosis management.

**Field-deployable sequencing.** The continued miniaturization and cost reduction in sequencing devices, such as the Oxford Nanopore MinION and forthcoming palm-sized sequencers, will enable real-time genomic surveillance in remote endemic regions. While conceptually appealing, mobile phone-based bioinformatics for NGS data remains largely experimental and faces substantial technical limitations, including limited computational power, battery life, and data transfer speeds in remote settings. More practical near-term approaches include cloud-based analysis platforms, which require stable internet connections that may not be universally available, or portable computing devices with pre-loaded analysis pipelines. The establishment of regional sequencing hubs, where samples can be referred for analysis, currently represents the most feasible model for translating genomic data into actionable public health information in resource-limited settings.

**Integration of artificial intelligence and machine learning.** While well-curated AMR databases such as ResFinder and AMRFinderPlus already enable genotype-based resistance prediction for known resistance determinants, AI/ML models may offer three additional advantages: (1) detection of novel resistance mutations not currently captured in curated databases, (2) integration of multiple genomic features (e.g., SNPs, gene amplification, efflux pump expression) to improve predictive accuracy for drugs where resistance mechanisms are heterogeneous (e.g., trimethoprim-sulfamethoxazole), and (3) automated pattern recognition for outbreak detection from real-time sequencing data. However, the small size of existing *B. pseudomallei* genomic datasets limits the training of robust AI/ML models, and international consortia are working to aggregate sequences from multiple endemic countries to address this limitation.

**Global genomic surveillance networks.** Substantial progress in melioidosis genomics has already been driven by coordinated efforts of several key research networks. The International Melioidosis Network (IMN) has facilitated global collaboration and data sharing across endemic regions, with notable outputs including the first systematic global burden of disease estimate for melioidosis [41]. The Mahidol Oxford Tropical Medicine Research Unit (MORU) in Thailand, in partnership with the Wellcome Sanger Institute, has generated large-scale genomic datasets that have transformed understanding of *B. pseudomallei* population structure and transmission dynamics, as exemplified by genomic studies linking bacterial genetic variation to infection and environmental adaptation [42] and investigations of host–pathogen interactions [43]. The Menzies School of Health Research in northern Australia has been instrumental in linking clinical isolates to environmental reservoirs through prospective genomic surveillance, as exemplified by the Darwin Prospective Melioidosis Study, which has integrated genomic surveillance with detailed clinical and environmental metadata since its inception [26]. Recent high-impact studies have further highlighted the utility of genomics for understanding pathogen spread in relation to climatic and environmental factors. Coulon and colleagues demonstrated the power of hybrid sequencing approaches in resolving complex genomic rearrangements in *B. pseudomallei* [19]. Currie and colleagues have linked genomic data to environmental risk mapping in northern Australia [44,45]. Chiu and colleagues traced an urban melioidosis outbreak to contaminated water reservoirs, using WGS to guide public health interventions [46]. Building on these existing frameworks, we advocate for a WHO-led initiative to establish a “Global Melioidosis Genomic Surveillance Network,” with core functions including: (1) establishing a curated, open-access global *B. pseudomallei* genome database; (2) developing standardized protocols for sequencing and data sharing; and (3) implementing training programs for bioinformatics capacity building in endemic countries.

It is important to distinguish the different roles of NGS across the spectrum of melioidosis management. At the individual patient level, mNGS may be most useful for critically ill patients with negative cultures or suspected polymicrobial infections, but its routine application is unlikely to be cost-effective in most endemic settings given the availability of rapid PCR-based diagnostics (e.g., TTS1-orf2 qPCR). At the outbreak investigation level, WGS of cultured isolates has already proven its value and should be the primary focus of NGS implementation. At the population surveillance level, environmental metagenomics and genomic epidemiology can provide early warning of emerging clones and resistance patterns, supporting public health decision-making. The “streamlined options” discussed, including targeted NGS panels and regional sequencing hubs, are best suited to bridge the gap between reference laboratory capabilities and front-line diagnostic needs, particularly for resistance surveillance and outbreak detection rather than routine individual diagnosis.

**Validation of targeted NGS panels for routine use.** An important consideration is whether NGS-based approaches can compete with multiplexed nucleic acid amplification tests (NAATs) for routine diagnostics. For species identification alone, PCR-based assays (e.g., TTS1-orf2 qPCR, LAMP, CRISPR-BP34) are faster, cheaper, and more suitable for frontline use in endemic settings. The value proposition of NGS lies in its ability to simultaneously detect multiple pathogens, identify AMR determinants, and provide genomic data for epidemiological investigation, capabilities that are not achievable with simple NAATs. However, for AMR detection specifically, even targeted NGS panels face competition from novel PCR-based approaches. For example, ARDaP (Antimicrobial Resistance Detection and Prediction) was developed specifically to predict resistance genotypes from sequencing data, highlighting the complementarity of sequencing and PCR-based approaches. Recently, Withatanung and colleagues described a phage-based PCR assay targeting phage genes for melioidosis diagnosis, demonstrating that simple molecular assays remain highly competitive. Thus, NGS is best positioned as a second-tier tool for cases where PCR is negative despite high clinical suspicion, for comprehensive resistance profiling, and for outbreak investigation, rather than as a replacement for frontline NAATs.

**Evidence gaps and research priorities.** Several unresolved questions merit attention in future research. First, prospective clinical validation of mNGS for melioidosis diagnosis in endemic settings remains limited; multicenter studies comparing mNGS with conventional diagnostic workflows and measuring patient-relevant outcomes (time to appropriate therapy, mortality, antibiotic use) are urgently needed. Second, standardization of laboratory workflows (including host DNA depletion methods, library preparation protocols, and bioinformatics pipelines) is essential to reduce inter-laboratory variability and enable cross-study comparisons. Third, optimization of host DNA depletion strategies, particularly for blood and tissue samples with high human DNA content, would improve mNGS sensitivity. Fourth, development of genotype-phenotype resistance models for *B. pseudomallei* requires larger datasets of paired genomic and phenotypic susceptibility data, particularly for meropenem and trimethoprim-sulfamethoxazole. Fifth, formal cost-effectiveness analyses in endemic settings, considering both clinical and public health outcomes, are needed to guide resource allocation. Finally, the establishment of shared genomic databases and data-sharing frameworks would facilitate global surveillance and accelerate the translation of genomic insights into public health action.

**Host–pathogen interaction studies.** While studies specifically applying dual RNA-Seq to melioidosis are limited, transcriptomic analyses of *B. pseudomallei*-infected macrophages have revealed bacterial adaptation strategies during intracellular survival, including upregulation of type III secretion system genes and metabolic pathways [47]. In animal models, RNA-Seq of infected tissues has identified host pathways that correlate with bacterial burden and disease outcome. Future application of dual RNA-Seq to clinical samples could reveal how bacterial gene expression changes in response to host immune pressures during treatment, potentially identifying markers of persistence and relapse.

## 8. Conclusions

Next-generation sequencing (NGS) is fundamentally transforming melioidosis management through three synergistic roles: enabling rapid, culture-independent diagnosis via metagenomic NGS (mNGS); providing high-resolution outbreak tracing and source attribution through whole-genome sequencing (WGS); and facilitating predictive, comprehensive antimicrobial resistance profiling. Recent 2025–2026 studies have significantly expanded these capabilities, demonstrating the utility of metagenomics for direct pathogen genome recovery from contaminated products, the detection of imported cases in non-endemic regions, and the discovery of novel resistance mechanisms such as the A172T mutation and promoter mutations upstream of *penA*. Nevertheless, substantial barriers remain formidable in resource-limited endemic settings. These include high costs, a lack of standardization, and limited bioinformatics capacity.

Overcoming these obstacles will require strategic, sustained investment in pragmatic solutions: validated targeted sequencing panels for routine clinical use, field-deployable sequencing devices for remote areas, AI-powered analysis tools to lower the barrier for data interpretation, making genomic analysis accessible to non-specialists in endemic settings through automated reporting and simplified user interfaces, and a coordinated global genomic surveillance network to harmonize data and share best practices. While individual-level “precision medicine” through NGS remains a distant goal in most endemic settings, the most immediate and impactful application of genomics lies in population-level genomic epidemiology and public health surveillance. By enabling earlier outbreak detection, environmental source attribution, and tracking of resistance emergence, NGS can inform interventions that prevent cases at the population level. However, it is important to distinguish between the established and emerging applications of NGS. The strongest evidence currently supports whole-genome sequencing for outbreak investigation and genomic surveillance, where the value of high-resolution strain typing and source attribution is well documented. In contrast, evidence supporting routine metagenomic NGS for frontline clinical diagnosis remains more limited, with most studies demonstrating feasibility rather than clinical utility. Prospective multicenter trials, standardized analytical pipelines, and economic evaluations are still needed before widespread clinical implementation can be recommended. With sustained investment in pragmatic solutions such as regional sequencing hubs, standardized protocols, and capacity building, genomic surveillance can substantially reduce the burden of melioidosis, particularly by guiding public health responses and strengthening global health security against emerging infectious threats, while continued research will define the appropriate role of mNGS in individual patient care.

## Figures and Tables

**Table 1:** Comparison of NGS approaches for melioidosis diagnosis and investigation.

Approach	Description	Optimal Use Case	Key Advantages	Major Limitations	Performance Metrics
**Metagenomic NGS (mNGS)**	Untargeted sequencing of all nucleic acids directly from clinical specimens (blood, pus, sputum, tissue)	Critically ill patients with unknown etiology; culture-negative cases; polymicrobial infections	Unbiased, hypothesis-free detection; identifies all pathogen types; no culture required	High human DNA background ^a^; complex bioinformatics; higher cost; requires careful interpretation	Sensitivity ~50% in sepsis (combined with other methods) [8]; turnaround 24–72 h; cost ~$500–1000 per sample
**Targeted NGS (tNGS)**	Amplification or probe capture of specific genomic regions (e.g., 16S rRNA, resistance genes, species-specific markers)	Confirmation of *B. pseudomallei*; screening for known resistance determinants; resource-limited settings	High sensitivity for targets; low host background; cost-effective; faster turnaround	Limited to known targets; cannot detect novel genes or unexpected pathogens	Sensitivity ~85–90%; specificity ~92% [7]; turnaround 12–24 h [7]; cost ~$100–300 per sample [7]
**Whole-Genome Sequencing (WGS) from culture**	Sequencing of entire genome from cultured isolate (can be performed with short-read, long-read, or hybrid approaches)	High-resolution outbreak tracing; phylogenetic studies; comprehensive resistome/virulome analysis	Gold standard for strain typing and source attribution; complete genetic context; enables hybrid assembly	Requires prior culture (adds delay) ^b^; demanding bioinformatics; higher cost and infrastructure	SNP accuracy > 99.9%; turnaround 3–7 d (including culture); cost ~$200–500 per sample

Note: ^a^ Major challenge due to high human DNA content and complex clinical matrices. ^b^ Introduces a 3–7 day delay prior to sequencing. For complex genomes such as *B. pseudomallei*, hybrid assembly (combining short- and long-read sequencing) is often necessary to resolve repetitive regions and obtain accurate SNP differences for clonal characterization. Performance metrics are approximate and derived from published studies; actual values may vary by laboratory protocol, sample type, and sequencing platform.

**Table 2:** Comparison of NGS applications across the three pillars of melioidosis management.

Application Domain	Primary NGS Approach	Key Advantages	Major Challenges	Critical Considerations
**Diagnosis**	mNGS (direct from clinical specimens)	Culture-independent; unbiased detection; identifies mixed infections; 24–72 h turnaround [8,11]	High human DNA background; complex bioinformatics; high cost; variable sensitivity [7]	Best as second-tier test after negative qPCR/LAMP; requires host DNA depletion [13]; most useful in critically ill or culture-negative cases
**Epidemiology/Outbreak Tracing**	WGS (from cultured isolates)	High-resolution strain typing; source attribution; environmental reservoir linkage; ≤5 SNPs for cluster detection [17,18]	Requires prior culture (3–7 d delay); demanding bioinformatics; reference databases needed	Complements not replaces epidemiological investigation; hybrid assembly improves resolution for complex *B. pseudomallei* genomes [14,19]
**Antimicrobial Resistance Profiling**	WGS/targeted NGS (from isolates or directly)	Detects chromosomal mutations (*penA*, *amrR*); predicts resistance with >95% sensitivity/specificity for ceftazidime [20,21]; identifies novel mechanisms [22]	AMR less widespread than Enterobacteriaceae (<1–2% in naïve isolates); TMP-SMX resistance mechanisms heterogeneous [23]; validation needed for meropenem	Chromosomal resistance stable within lineages; ARDaP and ResFinder aid interpretation [20]; emerging carbapenemase genes (blaNDM, blaOXA-48) require surveillance [22]

mNGS, metagenomic next-generation sequencing; WGS, whole-genome sequencing; AMR, antimicrobial resistance; SNP, single nucleotide polymorphism; TMP-SMX, trimethoprim-sulfamethoxazole; ARDaP, Antimicrobial Resistance Detection and Prediction.

## Data Availability

No new data were created or analyzed in this study. Data sharing is not applicable to this article.
